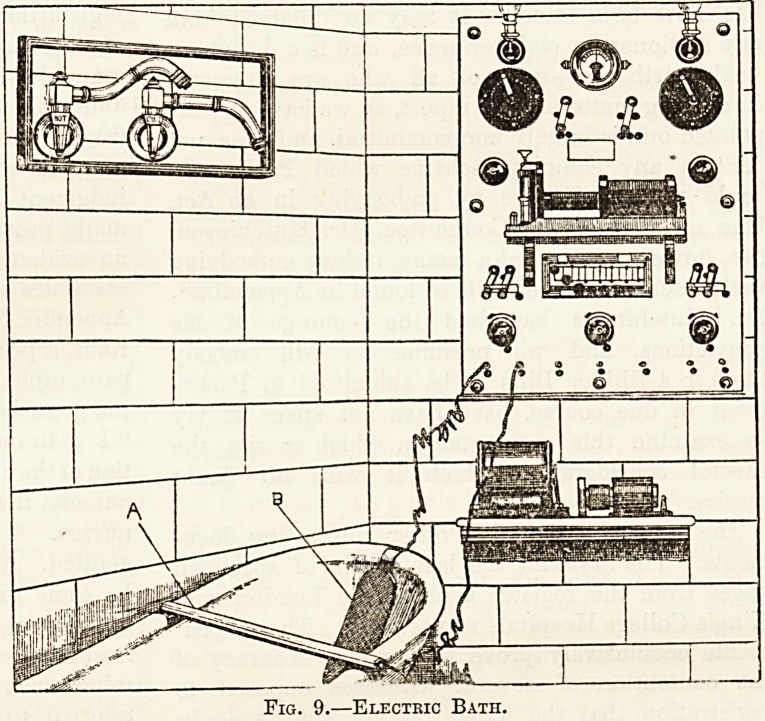# The Hospital. Nursing Section

**Published:** 1905-09-02

**Authors:** 


### The Hospital.

### Nursing Section.

Contributions for this Section of "The Hospital" should be addressed to the Editor, "The Hospital" Nursing Section, 28 & 29 Southampton Street, Strand, London, W.C.

No. 988.—Vol. XXXVIII. SATURDAY, SEPTEMBER 2, 1905.

### Notes on News from the Nursing World.

### THE QUEEN AND THE BRISTOL CHILDREN.

The Queen has sent to Miss Parry, of Olveston—who for more than 20 years has held a sale for the benefit of the funds of the Bristol Royal Hospital for Sick Children and Women—a parcel containing 12 baskets and six boxes of sweets, which her Majesty thinks may be acceptable to the little patients. Miss Knollys, in the letter accompanying the gift, mentions that she is forwarding from herself a quantity of back numbers of illustrated papers which may be of interest to the older inhabitants of the hospital. The president of the latter has suitably acknowledged both gifts, and has asked Miss Knollys to convey to her Majesty the warm thanks of himself and of the committee and managers for her kindness.

### LADY DUDLEY AND THE DISTRICT NURSES.

The visit by Lady Dudley to Burton Port, Annagry, and Arranmore, Ireland, in order to see for herself the work of the district nurses appointed as the result of the movement initiated by her Excellency, provoked a remarkable display of genuine enthusiasm. When the vessel containing Lady Dudley lay at anchor in Arran Roads there was a display of bonfires which illuminated the whole island, the inhabitants being anxious to do honour to her Excellency for placing on the island an efficient and highly - trained nurse. Leaving Burton Port, she next proceeded to Annagry, where she had a long talk with Nurse Brady, and congratulated her upon the success of her mission in the district. Subsequently she was conveyed by boat to Arranmore Island, and here a large number of inhabitants awaited her arrival on the beach in order to give her a hearty welcome. Lady Dudley spent some time with Nurse MacMahon on the island, and listened with keen interest to the account which the latter gave her of the progress of her labours. There is no doubt that her visit has satisfied the people among whom the nurses are working that Lady Dudley is personally solicitous to promote the better care of the sick and suffering.

### MRS. RICHARDSON'S WORK IN JAPAN.

Mrs. Teresa Richardson, on whom the Mikado recently conferred the Sixth Class Order of the Crown, and who has also been awarded the highest honour of the Japanese Red Cross Society—namely, the Order of Merit, has explained to our representative, in a special interview reported elsewhere, the precise nature of her work in Japan. She makes no pretence of being a trained nurse, but during the Boer War she nevertheless gained the South African Medal for the assistance which she afforded in the care of the sick and wounded, and she believes that her experience in South Africa was one of the reasons why she was able to be of service in Japan. But there were other reasons which apparently account for the fact that she was one of the few foreigners who were allowed to assist in nursing the Japanese soldiers. These were primarily, no doubt, the circumstance that she paid all her own expenses, which must have been considerable, and also her knowledge of European languages. For the rest, the interview of our representative with Mrs. Richardson entirely confirms all that has been said in our columns as to the absence of any necessity for the Japanese to employ European women, their own fully-trained nurses being perfectly competent to tend the wounded without the help of any outsiders.

### NURSING IN GAMBIA.

The Colonial Secretary for Gambia pays high tribute in his report to the excellent work of the sisters from the convent of St. Joseph of Cluny, in France, three of whom have for the last two years been employed as nurses in the hospital at Bathurst. This hospital is now one of the best on the West Coast of Africa, having been recently enlarged and put in thorough repair. A new wing has been erected for the female patients.

### THE DUTY OF SENDING FOR THE CHAPLAIN.

The Belfast Guardians at their weekly meeting had under consideration a report submitted by the committee who had investigated a complaint preferred against one of the nurses, of whom it was alleged that she had failed to send for the Roman Catholic chaplain when he had been asked for. The chairman of the committee stated the facts, and from these it appears clear that a request was made by a patient, who was dangerously ill and has since died, for the attendance of a priest, and that the nurse to whom it was preferred did not comply with it. Quite an unnecessary amount of acrimony was imparted into the discussion which followed Dr. Macintosh's statement, but we entirely agree with the course ultimately adopted by the committee, who instructed the clerk to write a letter of caution to her. This moderate reproof for action which cannot be justified, will, we hope, serve as a warning to her to remember that it is her duty to attend immediately to any request on the part of a patient seriously indisposed for the presence of the chaplain.

### INCREASE OF A MATRON'S SALARY.

It will be recalled that some little time ago Mrs. Richmond, the matron of Luton Workhouse, undertook the duties of superintendent nurse of the Infirmary. At the last meeting of the Luton Guardians the General Purposes Committee reported that, having considered the additional duties performed by the matron, they recommended that her salary be increased from £60 to £75 per annum. It was mentioned that Mrs. Richmond had performed the duties for 15 months, and that for three months they had had no additional help. The proposal was adopted unanimously, several of the Guardians expressing in cordial terms their appreciation of the manner in which the matron had fulfilled her obligations. We are glad that the advantages of having a trained nurse as matron of Luton Workhouse have been recognised by the Guardians.

### "CHANGE IN HOSPITAL NURSES."

The assertion is made by an ex-nurse in one of the daily papers that hospitals spend too much money, and one of the causes of this, she alleges, is that "nurses do so little for themselves." She says, "When I was a hospital nurse we nurses had to wait on ourselves. Now they have nurses' maids. When we went to cases we had our patients' room to keep clean. Now they want the servants of the house to do that." It may be admitted that there is a tendency on the part of some nurses to do too little for themselves. But it is inevitable that the elevation of the nursing standard should have been accompanied by such changes as an ex-nurse seems to deplore, and we rejoice that it is so. It is in the interests of good nursing, and therefore of the patients, that nurses in hospitals should, as far as possible, be relieved from work which can be done by domestic servants; and as to private houses, if people can afford to pay two or three guineas a week for the attendance of a nurse, there are usually servants in the establishment who, without additional expense, can keep the room of the patient clean. The true nurse does not consider anything she does for her patient degrading, but not even in order to effect economy in hospital administration, can we support an attempt to put back the hands of the clock.

### THE SITUATION AT SCULCOATES.

At their last meeting the question of the administration of the Sculcoates Union Infirmary engaged the attention of the Guardians. The report of the committee, which was submitted, was accompanied by that of two inspectors of the Local Gevernment Board who had attended their meeting. The inspectors stated that there was not the smallest particle of evidence forthcoming to justify any allegations of "ragging." They were, however, of opinion that the discipline of the infirmary had for the last two years been bad, and that the probationers had been insubordinate and troublesome. The allegations of harshness against one of the charge nurses had been very general, but they were probably, the report continued, very much exaggerated, and it was hard to see how a charge nurse, face to face with insubordination and unrest, could be expected to preserve her equanimity and keep her temper at all times. The attempt to introduce a religious animus into the controversy had not been in any way justified. Apart from cases of illness off duty time had not been materially interfered with. There was no direct evidence to show how any patients had been treated in any way but with extreme kindness by the probationers as far as was proper under the regulations. The committee were of opinion that the evidence showed that a charge nurse had been too severe in her dealings with the probationers, and that some of the head officials did not work together as they might do. They strongly recommended that if this state of things continued, the guardians would endeavour to ascertain who was to blame, and take steps to remove the cause of the friction. Finally, they advised the revision of the rules for the nursing staff. The guardians adopted the findings of the committee and rejected the report of the Local Government Board inspectors, by 23 votes to four. It will be seen that the situation at Sculcoates is still of a very unsatisfactory character; but if the reports of the inspectors and the committee are extremely conflicting, the conclusion that the rules of the nursing staff require revision is, at any rate, a sound one.

### NIGHT NURSING AT KIDDERMINSTER INFIRMARY.

The Kidderminster Guardians appear to have decided to ignore the recommendations of the inspector of the Local Government Board with regard to the system, or want of system, of night nursing. The inspector reported that the night nurse was not on the permanent staff, and was not a trained nurse. According to returns supplied to him, there was one night nurse to 164 patients, which was the highest proportion in his district for night nursing. He understood that the medical officer had mentioned the need of more supervision at night, and the undesirability of a female nurse having to cross from one infirmary to another, especially in winter weather. "Doubtless," he added, "the Guardians were actuated by a desire for economy, but the proper care of the sick was a precaution which, he could not but think, they would desire to exercise." The chairman, in referring to the report, said that as the Local Government Board did not ask to be furnished with the guardians' observations on the nursing accommodation question, it was not necessary to reply then to that; and the only notice taken of the inspector's allusions to the night nursing was a statement that the guardians were now paying a temporary night nurse £1 a week. The importance of raising the standard of nursing in Poor-law institutions is evidently not yet realised by the Kidderminster Guardians.

### THE QUESTION OF A MIDWIFE AT ENNIS.

The Ennis guardians have decided to try and set the Irish Local Government Board at defiance. The question of appointing a midwife for the dispensary district of Ennis having been several times discussed by the guardians without any result, the Local Government Board at length sent a sealed order directing that a midwife be appointed by the guardians at a salary to be approved by the board. A very good point was made by one of the guardians during the discussion on the matter. In supporting the proposed appointment, he said that "this nurse was one for the poor, and if they were going to debar the poor of the opportunity of getting her services, it would be doing them a great injustice." Nevertheless, the majority of the guardians—17 to 6—voted in favour of an amendment hostile to the appointment, and it will now devolve upon the Local Government Board to take measures to show in a manner which cannot be mistaken that the supreme authority must be obeyed.

### NURSE AND MASSEUSE.

A letter from a correspondent on the subject of the "Nurse and the Masseuse" a few weeks back has been followed by a further communication which appears in another column this week. Both writers complain that, having qualified themselves at the cost of time, trouble, and expense for the treatment of patients by massage, they now find their work to a certain extent taken away from them by nurses who, though not having any special qualifications, are willing, so as to secure an appointment, to "throw in" such massage as their general training and all-round experience enables them to do. But this is not as it should be. If a woman in addition to her other training goes in for massage there is no reason why she should not give it if her patient and the medical man desire it, though it is doubtful whether a nurse tired with disturbed nights or the strain of nursing all day is likely to be as successful as a woman coming straight to her case from outside, whose whole mind is bent upon massage in its various forms. But if the only qualification is a desire to be "obliging" a nurse has no right to practise massage for two reasons. In the first place because the attempt is likely to do her patient more harm than good; and secondly because it is unfair to those who have paid money to acquire an art which will gain them a livelihood. Nurses would be justly indignant if a masseuse undertook to do general nursing. How is it that some of them fail to see the case in the same light when the positions are reversed?

### THE MILITARY AND NAVAL SERVICES.

Miss Eva Catherine Ellis has been provisionally appointed staff nurse in Queen Alexandra's Imperial Military Nursing Service; and Miss C. A. Whyham has been appointed a sister in Queen Alexandra's Royal Naval Nursing Service.

### QUEEN'S NURSES AT NORMANHURST.

Through the kindness of Lady Brassey, the nurses of the Sussex County Nursing Association had their first social gathering at Normanhurst on Wednesday, August 23rd, when a most enjoyable afternoon was spent. Those coming from a distance were met at Battle and Bexhill stations and driven to Normanhurst, where they received a hearty welcome from Lady Brassey who afterwards entertained them to tea. The Hon. Mrs. C. A. Egerton (hon. secretary of the Association), after expressing regret at the unavoidable absence of Miss Hughes (the newly-appointed superintendent of the Queen Victoria's Jubilee Institute for Nurses), read a paper sent by her dealing with the difficulties, possibilities and responsibilities of the nurses. In the course of a short address Mrs. Egerton placed before the nurses a high ideal, and urged them not to be discouraged by the difficulties they had to encounter, reminding them that the success of the Association depended not so much on the managing committee and subscribers as on the patient, kind, and skilful work of the nurses themselves. The remainder of the afternoon was spent in strolling about the beautiful grounds, and when later the nurses left for their respective districts it was with memories of a happy and profitable afternoon spent at Normanhurst. Besides the nurses and county superintendent several ladies of the county and local committees were present, thereby testifying the interest taken by them in the nurses and their work.

### VICTORIAN TRAINED NURSES' ASSOCIATION.

Two interesting statements are made in the annual report of the Royal Victorian Trained Nurses' Association. First, that although the Association was the first to adopt the policy, "admittedly ideal," of making all branches of special training depend upon the possession of a general certificate, owing to the dearth of registered applicants, a few probationers have had to be employed, who, however, will not be given a certificate until they have a place on the general register. Moreover, a special conference has been convened to discuss the question of obstetric nursing. In the opinion of the council the best and only advisable way of obtaining efficient obstetric nursing for all is to educate the public to a better understanding of what are, and what are not, the real duties of the nurse; to encourage medical men and nurses to mutually co-operate along the lines of the visiting nurse; to extend the operations of the District Nursing Association; and, above all, to maintain the efficiency of obstetric nursing. The council asserts its belief that under the Victorian conditions such an Act as the Midwives Act of Great Britain "is not only unnecessary but would do much more harm than good."

### DANCES AS A SOURCE OF INCOME.

At the annual meeting of the Grantham Victoria Nursing Association, the report of the committee was read and adopted. There is one passage in it which we desire to endorse. The committee, while characterising the state of the finances as fairly satisfactory, affirm that they cannot be said to be so altogether as long as the funds are largely dependent upon the annual dances. These they consider a too unstable source of income, and they are quite right. Even as it is, the income only exceeded the expenditure by less than £2, and therefore, without the money which the dance yielded, there would have been a serious deficiency. The mainstay of the association should, of course, be the subscriptions, and it will be a great mistake to regard contributions which might at any time cease to be received, in the light of a certainty. In any event, the amount is sure to vary; and, moreover, it would be fatal to the efforts of the collectors to encourage the idea that the organisation has always the proceeds of a dance to rely upon. It is very pleasant and desirable to have assistance of this kind, but a nursing organisation had almost better be without it than regard it as the backbone of its income.

### The Nursing Outlook.

" From magnanimity, all fear above;
From nobler recompense, above applause,
Which owes to man's short outlook all its charm."

### THE REGISTRATION OF NURSES.

The report of the Select Committee on the Registration of Nurses, together with its proceedings and the minutes of evidence and appendices, has now been issued. It may be obtained from any stationer for eighteenpence, and is a document well worth the study of all who are interested in nursing matters. The report, as we have already pointed out, is largely non-committal, and does not contain any complete scheme which Parliament could render effective by embodying in an Act. One member of the Committee, Dr. Hutchinson, has, however, prepared a memorandum embodying such a scheme, which will be found in Appendix 8. Dr. Hutchinson has had the courage of his convictions, and we presume he will embody them in a Bill or Bills to be submitted to Parliament in due course. We have not space to-day to examine this memorandum which merits the careful consideration which it will no doubt receive.

The appendix contains other interesting documents. The first and the last consist of specimen pages from the register kept at the London and King's College Hospitals respectively. These documents conclusively prove the precise accuracy of the contention of several witnesses opposed to registration, that the nurse-training schools do in fact keep a very complete and accurate register of the training and conduct of the probationers, sisters and nurses during their residence at the hospitals. These registers also contain information concerning the work and history of the nurses, who have received certificates, subsequent to their leaving the institutions where they were trained. Now that every nurse-training school of any importance has fallen into line with the three years' course, and has set up a register similar to those which will be found in the appendix, it should not be difficult, if the nurse-training schools will co-operate, to organise a system, whereby a general register of all the efficient nurses at present engaged in this country can be speedily prepared and published, under the auspices of a committee of the nurse-training schools. The House of Commons Committee have prepared the way for such a step by recommending, that the central body, appointed by the State to keep a register of nurses, shall admit to it all nurses who have been trained at a recognised training school, who have obtained a certificate from that school that they are equipped with the knowledge and experience requisite for nursing, and that they are of good character.

The third appendix consists of a paper handed in by Mrs. Bedford Fenwick, headed "Some Typical Nursing Scandals." This contribution embodies eighty-nine of these so-called "typical nursing scandals," each of which "was reported in and extracted by the witness from a public newspaper." The Committee apparently refer to this document and the evidence based upon it, in the following paragraph of their report: "It has been asserted in some quarters that registration is rendered requisite by reason of the amount of illegality, immorality and scandal which at present continues undiscovered and unchecked. It is contended that registration would be an efficient instrument against these scandals and would safeguard the public. In the judgment of your Committee, while registration might prove a means towards checking some abuses, no evidence which has been brought forward substantiates a general charge of moral delinquency." Appendix No. 4 consists of a paper by Miss Beatrice Kent, a private nurse, which contains the following paragraph giving expression to the views held by the great body of reputable nurses on this question: "I wish most emphatically to oppose the suggestion of the chief witness at one of the recent meetings, namely, that there is so much criminality among nurses. I consider the statement greatly exaggerated. We are not a criminal class. There may be some examples of grievous wrong-doing, I do not deny it, as there are black sheep in every fold. Accounts culled from newspapers are not always reliable; it is more than probable that these women referred to were not nurses at all; thieves and swindlers often adopt the dress of clergymen and call themselves reverend. I consider the matter irrelevant. State registration will correct abuses of course, because it will call forth a new order of things, but it has nothing to do with criminality." That is surely all that need be said on this subject.

The remaining appendices contain information showing the nursing officials, nurses, private nurses and probationers employed in 499 hospitals, and Poor-law infirmaries of 50 beds and upwards in January 1905, together with the figures showing the yearly average vacancies and the yearly applications for training at these institutions; tables showing the cost of nurses during 1903 at the principal London general hospitals and at hospitals in the provinces and Scotland with upwards of 200 beds from which the information is procurable; the rules and regulations issued under the authority of the Secretary of the Board of Supervision for regulating trained sick nursing in poorhouses in Scotland; particulars of training and application for admission to the examination for the nursing certificate of the Medico-Psychological Association; and an interesting memorandum by Dr. Harding on the system of training mental nurses pursued at the Northampton County Asylum. This blue-book should certainly be studied and preserved by nurse-training school authorities and nurses generally.

### Medical Electricity and Light Treatment.

By Kate Neale, Sister-in-Charge of the Actino-Therapeutic Department, Guy's Hospital.

### VI.—ELECTRIC BATH.

One of the difficulties encountered when treating by galvanism or faradism in the manner previously described, is the great resistance the skin offers to the passage of any current. This drawback can, to a great extent, be overcome by using a wet electrode which serves to moisten the skin and so diminish its resistance, but the same effect can be produced more certainly by surrounding the whole limb, or, if necessary, the entire body with water through which a current is passed. In this way a very intimate contact is provided between water and skin, and the resistance is lowered to a minimum. There is yet another advantage the "electric bath," as it is called, possesses, and that is, that it provides a simple and efficient means of administering treatment to the whole body at once. This is of great service when many different areas or joints have to be treated, or when a stimulus to the body generally is required.

The doctor may direct you to treat in one of the following ways:—1. Whole electric bath; 2. Local bath; 3. Electric douche.

The three varieties are easily understood. In the first the patient is completely immersed in water except for his head, but in the second only one limb or part of a limb is submerged. The electric douche differs from the two preceding in that no part of the patient is placed in a bath, but a stream of water, charged with electricity, is played upon his skin.

### I.—Whole Electric Bath.

**Apparatus.**

The bath itself is usually made of porcelain and calls for no special comment. Care has to be taken that its connections with the earth are efficient; this, however, is a matter that concerns the doctor rather than the nurse. Laid across the top of the bath is a metal bar (fig. 9, a) to which is connected one of the battery wires, thus converting it to an electrode. The bar is movable and can be slid to and fro between the head and foot. Inside the bath at the head is the other electrode b. This is a large metal plate, 21 × 8 inches, movable, and curved to adapt itself to the slope of the bath. Its upper end is prolonged to hook over the edge of the bath, and is provided with a binding-screw to which the second battery wire is attached. The patient grasps the bar electrode with both hands so that the current coming in, let us say, at the head electrode, passes through the water to his body, and finally runs down his arms to the bar, and so back to the battery.

In some cases a second plate-electrode put in the water at the foot of the bath is substituted for the bar. The current then enters at one electrode, runs through the water and passes out at the other, and only a small portion of it traverses the patient. With this arrangement a much stronger current can be used than with a bar electrode when the whole of the current flows through the patient's body.

You will remember the warning I gave you earlier

Fig. 9.—Electric Bath.

to protect the skin from contact with bare metal. The same care must be observed in the case of the bath, and the safest method is to cover the plate electrodes with a rubber mat about an eighth of an inch thick, and perforated with many holes. The bar electrode may be left exposed, for it is touched only by the palm of the hand, and there the skin is thick enough to be sufficient protection.

If the bath used is insulated, the greatest care must be taken to prevent any possibility of the patient coming into contact with the pipes connected to the earth. To this end, both hot and cold water taps must be placed well out of his reach. At Guy's Hospital the further precaution is taken of enclosing the taps in a glass case provided with lock and key.

As regards the varieties of electricity employed for an electric bath, galvanic and faradic currents are often ordered, but I need add nothing to the account of them already given.

There is, however, a third form, which we have not met up to now, and this will require some brief attention. It is known as the **Sinusoidal Current,** and is in some respects similar to a faradic or interrupted current. It consists of a number of brief electric currents following each other with great rapidity; but instead of being suddenly made and suddenly broken, as with an induction coil, they gradually rise to a maximum and then as gradually die away. The result of this difference is that the sharp pricking feeling of the faradic current is abolished, and we have instead a gentler and smoother stimulus. A special motor is used to produce the sinusoidal current, but the method of application resembles that of faradism.

**How to Treat.**

While the bath is being administered it is essential that someone should be present with the patient the whole time to guard against accidents; and therefore, if the doctor wishes, you must be ready to remain throughout the treatment. Connect the battery wires to the binding screws on the head and bar electrodes, or on the head and foot electrodes, according to which have been ordered, and fill the bath with water to a height sufficient to reach well up the plate electrode, and at the same time to cover the patient. The water must be hot, between 90° and 100° F., but, as a rule, should not exceed 99°, and in hot weather the temperature may be nearer 90°. Never rely on the rough estimate of the temperature made by the hand, but employ a thermometer. When enough water has been run in, take care that the taps are moved out of the patient's reach, and if there is a case to enclose them, see that they are safely shut away. The last precaution you must observe before the patient enters the bath is to test the full strength of the current on yourself. This must never be omitted, and can be done by placing one hand in the bath while the other grasps the bar electrode.

These preparations made, the patient, garbed in an ordinary bathing dress, enters the bath. Allow him a few minutes in which to become accustomed to the water, and then, directing him to grasp the bar with both hands, switch on the current gradually until the proper strength is reached. If the whole force of the current be suddenly brought into action, the effect on the patient may be alarming. It is impossible to specify the exact strength that should be used, but the doctor will decide this in each case, and you must be careful to follow his instructions; but as a general principle you should never administer a current strong enough to be disagreeable. It is not essential for the patient to be in actual contact with either the head or foot electrodes, and if an interval of a few inches is allowed there is perhaps less risk of any harm being done to the skin.

Continue the treatment for from ten to twenty minutes, and then, before the patient leaves the water, gradually switch the current off. Let him briskly dry himself (unless by rheumatism or other disease this is impracticable, when assistance must be given), and, after dressing, let him rest, protected from draught, for a quarter of an hour or so before leaving. The bath is not as a rule repeated more frequently than every other day, and it is hardly necessary to warn you against giving one soon after the patient has had a meal.

II.—Local Bath.

This form of bath differs only slightly from the one just described. When the upper limb is to be treated a special arm bath is required, and an electrode shaped somewhat like a raquet is placed at the bottom, and the handle part, bent to lie against the side of the bath, reaches above the surface of the water and ends in a binding screw. A perforated sheet of india-rubber covers the expanded portion to prevent contact with the skin.

**How to Treat.**

Fill the bath with water at a temperature of 99°, and place the electrode in position with one battery wire attached to its binding screw. Wrap the other battery wire in a sponge and press it as an indifferent electrode against the nape of the neck as described under Galvanism (p. 254). Direct the patient to put as much of his arm as possible under water, resting the hand on the rubber mat, and then gradually switch on the current after having tested it on yourself. Sinusoidal, galvanic, or faradic currents may be used, and a single treatment lasts from ten to fifteen minutes. Again, remember to switch off gradually before the patient lifts his arm out of the bath.

In the case of the leg a specially shaped bath is used, and treatment is applied in every way as with an arm bath.

III.—Electric Douche.

This method is much less frequently used than either the whole or local bath, and a few words of description will suffice. The douche may be given in one of two ways. A large indifferent electrode (such as is seen in fig. 2, c, p. 268) is connected with one wire from the battery or coil and brought in contact with the patient. The other is joined to an insulated metal nozzle from which a stream of warm water can be directed against his skin. The water in flowing from the nozzle becomes charged with electricity which is thus conveyed to the patient. In the second method, each battery wire is joined to an insulated nozzle, and two streams of water charged with electricity are played on the skin.

Whether the patient undresses wholly or partially depends of course on the site of application of the treatment, but if either trunk or leg be treated it is advisable for him to stand in a bath into which the stream of water can run.

**Dangers of the Electric Bath.**

If you always make a practice of testing the current on yourself before applying it to the patient, and also take care to prevent him coming in contact with any metal pipe or tap, you should not meet with any serious results from bathing. Sometimes the whole bath will cause a headache, but this can be relieved by some simple remedy such as a damp towel to the head. In other cases an over vigorous current may produce faintness, in which event the strength must be moderated. The effect of the current on any sore is painful, and such areas must be protected before the patient enters the bath. Finally, if the metal electrodes are left uncovered by rubber or other protective material, injury to the skin may follow.

**Diseases Treated.**

Treatment by baths is often ordered to cases of joint disease such as rheumatism or gout, and you will also be called on to treat sciatica, lumbago, lead palsy, paralysis, etc. Of all diseases for which the local bath is prescribed perhaps writer's cramp is the commonest, though the course of treatment may run into many weeks. Feebleness of circulation as seen in Raynaud's disease, and in people subject to chilblains is often improved by local or general bathing.

### The Nurses' Clinic.

### THE DISTRICT NURSE AND SANITARY LEGISLATION. BY A SUPERINTENDENT OF DISTRICT NURSING.

In the district nurse's daily routine the questions often arise—What is the law on this point? Is there any inexpensive way of putting the law in motion? Can I do it without drawing unfavourable attention to the work of the local Nursing Association?

The principal Act of Parliament which concerns all workers in the houses of the poor is briefly known as the Public Health Act, 1875. (1) A few clauses have been repealed and many additions have been made, notably in (2) the Canal Boats Acts of 1877 and 1884; (3) the Public Health (Water) Act of 1878; (4) Housing of the Working Classes Acts, 1885, 1890 and 1903; (5) Infectious Diseases (notification) Act, 1889; (6) Infectious Diseases (prevention) Act, 1890; (7) Factory and Workshop Acts, 1891 and 1901; (8) Employment of Children Act, 1903.

The full text of these can be studied in the reference room of most public libraries, but the leading provisions of each, as far as district nurses are concerned, are:—

1. No house, either in town or country, may be built without drains; and those originally constructed in that way must be altered. In cases where the use of cesspools is permitted they must not be under any part of the dwelling and must be properly cemented and ventilated. No house may be built or rebuilt without a sufficient water-closet, earth-closet, or properly constructed privy and ashpit. If the local authorities undertake to remove house refuse they must do so at regular and frequent intervals. If any house is proved to be without a proper water supply the local authority can compel the owner to provide it. Cellar dwellings are illegal, with the exception of those lawfully occupied in 1875. They must be at least seven feet in height in every part, and at least three feet of the height must be above the surface of the adjoining street. The cellar must also have an open area of at least two feet six inches wide extending along its entire frontage, must be properly drained, must have a proper chimney or flue, must have a window of at least nine clear superficial feet, and must be provided with a properly constructed place of convenience.

The following are all illegal nuisances, and redress can be obtained on complaint:—

Any house in a state injurious to health. Any house or part of a house so overcrowded as to be dangerous or injurious to the health of the inmates, whether or not members of the same family. Badly-kept animals. Any gutter, ash-pit, privy, etc., allowed to remain in a state injurious to health of inmates of the house or adjoining houses.

2. Every canal boat must be registered, and must be used as a dwelling only for the number of persons of the age and sex for which it is registered. Children on canal boats are subject to the Elementary Education Acts of 1870, 1873, 1876, and 1880.

3. Every occupied dwelling-house must have a sufficient supply of wholesome water for consumption and domestic use of the inmates "within a reasonable distance." It can be gathered from another paragraph in the Act that 200 feet is considered a reasonable distance, but in rural districts many people still have to send half a mile or more for anything resembling "wholesome water."

4. This Act brings "a tent, van, shed, or similar structure used for human habitation" under the nuisance provision clause of the Act of 1875.

5. The "infectious diseases" to which this Act applies are small-pox, cholera, diphtheria, membranous croup, erysipelas, the disease known as scarlatina or scarlet fever, and the fevers known by any of the following names—typhus, typhoid, enteric, relapsing, continued or puerperal. The notification must be made to the Health Officer of the district by the head of the family to which the patient belongs, or the nearest relative present in the building, or "the person in charge of or in attendance on the patient," or in default of these by the occupier of the building.

6. This Act applies to every "structure used for human habitation." Its chief provisions are:—

(1) Compulsory cleansing and disinfection of any premises or of any articles when such a course of action "would tend to prevent or check infectious disease."

(2) Persons ceasing to occupy a dwelling in which any person has within the previous six weeks suffered from any infectious disease are bound either to disinfect the dwelling to the satisfaction of a registered medical practitioner, or to give due notice to the owner of the dwelling. Any person failing to do so, or knowingly making false answers on being questioned by the owner of the dwelling or by any person negotiating for the hire of the dwelling, shall be liable to a penalty of £10. Any person placing, or allowing to be placed, any infected rubbish into any ash-pit or other receptacle for refuse without previous disinfection, is guilty of an offence under this Act.

7. Every factory must be kept in a cleanly state, must be free from effluvia arising from any nuisance, must be provided with a sufficient number of places of convenience for both sexes, must not be so overcrowded as to be injurious to health, must be ventilated in such a manner as to render harmless, as far as practicable, all gases, vapours, dust, etc., caused by the work. When the conditions of the work make it necessary the floor must be adequately drained. All children employed under 13 years of age and all children under 14 who have not a labour certificate, are subject to the Elementary Education Acts. The period of employment, exclusive of meal hours and absence from work, shall not exceed 14 hours for women, 12 hours for young persons, and 10 hours for children on any one day; and in any one week must not exceed 30 hours for children, and 60 hours for women and young persons. None of these may be employed continuously for more than five hours without an interval of at least half an hour for a meal. Women in laundries may work overtime, but not more than two hours in one day, or more than three days in one week, or more than 30 days in any year. Unfortunately, these provisions do not apply to small laundries employing only members of one family living together and "not more than two persons dwelling elsewhere." It is precisely with these that the nurse is concerned, but the historic ghost of the "widow with nine small children totally dependent upon her" guards the threshold.

The general restrictions on the employment of children, are:—

A child shall not be employed between the hours of nine in the evening and six in the morning; a child under 11 may not be employed in street trading; no "half-timer" at a factory shall be employed in any other occupation; a child shall not be employed to lift, carry, or move anything so heavy as to be likely to cause injury to the child, nor in any occupation likely to be injurious to his life, limb, health, or education.

Local authorities can and do make by-laws regulating the employment of children, and the nurse should acquaint herself with those affecting the district in which she works.

Information as to any breach of these laws, or suspected breach of them, should be sent to the medical officer of health, or to the sanitary inspector, and may, if the nurse chooses, be anonymous.

### Incidents in a Nurse's Life.

**Contributions for this column are invited.**

### MY FIRST CASE.

How many of us, on leaving the strict life of hospital, look forward with pleasurable anticipation to the "home" life of private nursing. I know I did. I imagined how I would gradually overcome all the popular prejudices against a hospital-trained nurse by my tact, forbearance, and adaptability. It should never be said of me that I turned a house upside down, and that the occupants were thankful when I left. All the tirades against the impossible ways of a trained nurse in a quiet, private house were fermenting in my brain, and everything they did I was not going to do.

I looked forward with longing to seeing my patient gradually gaining health under my care and mine only. I imagined the grateful relatives and friends looking up to me with admiring eyes. Perhaps it has been so since, but alas for my first case. My blood boils even yet when I think of what I suffered during that dreadful time.

I was sent to a solemn, impressive-looking house in the West End of London. I had been warned that it was not an interesting case; but even the prospect of an old man, a chronic hernia, did not damp my enthusiasm.

The cab stopped, I got out, pressed the bell, and waited with a wildly-beating heart. I was shown up a magnificent staircase into a small room. "The Nurse's Room." It contained two straight-backed chairs, a small table, and a chest of drawers with glass, no bed, as I was quick to note. I was relieving another nurse who had broken down. I remember wondering why she had done so, with only one patient. I know now. We exchanged a few words and I asked her what the treatment was.

"Treatment," she said laughing, "there isn't any. He orders his own treatment. You will not get a bit of peace. I am worn out."

I passed by her words lightly, not placing any real value on them, and after arranging my cap and apron, she took me into the next room to be introduced to the patient. In an invalid chair by the fire sat an old man. Even to my inexperienced eyes, he had a terrible expression of arrogance and selfishness. I said, "Good evening."

"Oh! you've come," he said, in a harsh, rasping voice. "I see I shall have to teach you how to talk to me. Come close here. Put your mouth to my ear and shout."

I did; and such was my mode of conversation for nearly three months.

"Put my feet on the stool," he shouted in a few minutes. He never spoke in an ordinary voice—always a bellow. I complied.

"I am very ill, nurse," he continued. "Very ill, indeed. Feel my pulse. Take it," he roared. "Where's your watch?"

I did so and found no cause for alarm. It was much more regular than my own at that moment.

"What is it?" he asked.

"Sixty-six."

"I don't believe it is," he went on, and to my great surprise he took out his own watch. "Sixty-eight," he announced triumphantly. "I knew you were wrong."

I was silent from sheer amazement. Presently he thought he would like some medicine and sent me into the next room for some. I found the bottle and a measure, and before pouring it out, asked when he had had it last.

"Now, nurse," he replied "let us understand each other from the beginning. I'll have no hospital nonsense here. If I want medicine, I'll take it, and if I don't, I won't. Give me the bottle."

"But"—— I began in dismay and then stopped. He snatched the bottle out of my hand, and tossing the cork on the floor, took a long draught. He then handed it back to me with a grim chuckle at my expression.

"I can see you are fresh from hospital," he announced. "I've had them before. They are good nurses when they leave here. It is quite an education to nurse me."

It was, as I soon found out. Presently a lady, over-dressed and fashionable came in, and after inquiring about the patient's health, and receiving the answer, that he was sinking fast, she turned to me and told me my duties. I was to rise at seven o'clock, dress and give the patient a cup of coffee, prepared over night, then assist him to wash. At 8.45 I was to go down to the kitchen and bring up his porridge, and then at 9, go down again for his breakfast tray. I was to give him his breakfast and then come down and take my own with the members of the family.

For the rest, I was to sit in his room, and do whatever he wished; write notes, rub his ankles, help him in and out of bed. At 11 I was to carry all his belongings into the next room. These consisted of two air-cushions, an ear-trumpet, flask of brandy, medicine bottle, rug, stick, and newspapers. Then I was to take him in and "settle" him in an arm-chair by the fire; "sit with him again until 1 P.M., then go down and bring up his lunch. I was to give it to him, and get my own; be off duty from 3 to 4.30; at 5 make tea for myself and patient; then clear away and wash up in the next room.

"He begins to go to bed at 10.30," she concluded with. "But I shall show you to-night. You quite understand that you sleep in his room, don't you?"

I did not understand anything of the kind, but rose to the occasion and said, "Oh! certainly."

"You won't get much sleep," she said pleasantly. "Dear papa has such bad nights, but you nurses are trained to do with very little sleep, aren't you Of course, if you have three or four very bad nights I don't mind letting you have an hour during the day."

She paused, evidently expecting me to be overcome with gratitude, so I thanked her and departed downstairs for the dinner-tray. It was nearly 12 o'clock before I lay down on the narrow little bed in my patient's room, throbbing with fatigue and vaguely conscious of a dawning disgust. We began about 10, and after an hour's terrible groaning, managed to get him ready. He had to be propped up with innumerable pillows, in case he got a heart attack, he said. Then, just as I was thinking of returning to my little room and undressing, he half opened his eyes and said, "Rub my ankles, nurse."

For nearly an hour I rubbed them. Every time I showed any signs of stopping, he bade me go on, as he liked it. At length when I felt ready to cry, he ordered me to get undressed and "Be quick."

Shall I ever forget that night? The stuffy room. Gas full on. Temperature about 70°; gas-fire blazing, not a window open; and that awful figure sitting up in bed with half-open eyes. Even if I had dared to go to sleep I could not have done so; as, when he was awake, he groaned and grunted and talked to himself; and, when asleep, snored like a multitude of pigs. Towards morning I fell into an uneasy dose, but was soon awakened by a voice demanding beef-tea.

With the knowledge and experience I now possess, I can see that I was simply an attendant. Any untrained person could do what I did. Only, as my patient knew well, no untrained attendant would do day and night work. He genuinely believed he was seriously ill, and imagined no one in the world understood his peculiar complaint but himself. Really it was simply age that was the matter with him. At all hours of the day and night the doctor was telephoned for, and generally I was sent out of the room during his visit.

Sometimes he would come out with a broad grin on his face.

"I am dismissed, nurse," he would say. "Transgressed beyond forgiveness." Then, before he reached the foot of the stairs, I was sent flying after him, and a reconciliation took place.

For more than two months I lived the same monotonous life, my only companion this old man. He delighted in telling me long tales of his wild youth and early life, spent in India amid scenes of debauchery and license. He seemed to delight in watching my blanched face as he unburdened his evil mind, glorying in his own wickedness. Of course, I know now that I was foolish, too anxious to please, and timid. If I had taken a firm stand, insisted on proper off-duty time and proper treatment, things would have been different. But, fresh from hospital, my head full of matron's admonitions, anxious to avoid friction, I erred too much on the right side.

Of religion, my patient had none. He would speak with great contempt of Christians, and ridiculed my efforts to vindicate my faith.

"Do you suppose, nurse," he said one day, "that God wants to be bothered with such as you? If there is a God, He doesn't want your gratitude. You Christians sicken me. If I had done anything for a person I would not want them to be everlastingly on their knees thanking me. I believe there is a Supreme Power, but I do not believe that He takes an individual interest in each of us, as you seem to think. The whole thing is absurd."

Such arguments always silenced me, and after a few weeks I began to wonder if anything was real and true. Everything seemed to be slipping from my grasp.

One evening, after eating an enormous meal—he had been warned against such heavy, rich food—he had an apoplectic seizure. The doctor was hastily summoned; he did not come immediately, thinking, no doubt, that it was the oft-repeated cry of "Wolf!" There was no hope; four hours after the attack he died. Never shall I forget that death-scene. It is a landmark in a life of experiences. The brilliantly lighted room, the relations all standing round the bed, and the heavy, inert, figure, the last convulsive breathing.

A few minutes before he died he regained consciousness, and, looking round in a vague, frightened way, very unlike his usual glare of defiance, said, slowly and with many pauses. "I—think—it—would—have—been—better—if—I—had—thanked—Him."

A few seconds more and the darkened soul went into the presence of Him who understands and forgives.

I have nursed patients since then whom I have loved sincerely, but I have never shed such bitter tears as I did over my first. It was all so hopeless and sordid. The skill and knowledge which perhaps I secretly prided myself on were not needed. Just a patient endurance and a boundless tact. My dreams of being a ministering angel to an admiring family were shattered. I was "Papa's Nurse," the much enduring attendant of a domineering, bad-tempered old man.

### Nursing under the Japanese Red Cross Society.

### INTERVIEW WITH MRS. RICHARDSON.

Mrs. Teresa Richardson, a Welsh lady, widow of the late Mr. John Crow Richardson of Glanbydan, Carmarthenshire, who has for the last fifteen months been working with the Japanese Red Cross Society, arrived in England last week, and kindly accorded an interview to our representative.

At the outset Mrs. Richardson said that she was extremely averse to publicity, and would have rejected all the advances of the Press if she had not felt that people ought to know of the excellent organisation of the Japanese military hospitals. She stated that she was about to publish a book giving details of her experience.

#### Not a Trained Nurse.

Mrs. Richardson said that she was not a trained nurse, properly so called; she had never lived in a hospital, but had attended in the mornings for some time. But this was many years ago, and she had refreshed her nursing knowledge by attending enteric fever cases at Bloemfontein during the South African War, for which work she had obtained the South African medal. She had gone out to Japan in order that she might help the Japanese nurses. It was not true, as she had seen stated, that she had undertaken work of a special nature. She had at first only assisted the nurses, and later had fulfilled all the usual duties required. The only way in which her position might be considered special, was that she had paid all her own expenses. Apart from the fact that to keep a European nurse would cost about four times as much as to keep a Japanese, it was not at all necessary for the Japanese to employ European women as their own nurses were admirably capable. She was the only foreign woman nursing the soldiers, with the exception of a party of Americans who came out for six months and left last October, and a sister attached to two surgeons of the German Red Cross Society who arrived in Japan this year. She believed that if it had not been for her South African experience and her fluent knowledge of French and German, she would probably not have been of service to the authorities.

#### The Arrangements for the Sick.

Mrs. Richardson referred to the splendid conduct of the ladies of the Japanese nobility, who, after their tremendous task of rolling bandages was completed, used to give so many mornings a week to hospital work. With regard to the Japanese soldiers as patients, she said that they were heroic, and delightfully courteous; and, as she expressed it, "the most sweet-tempered of people." She had never heard any bickerings or quarrels between the men. She was enthusiastic over the excellent arrangements for the sick. No women nurses were allowed to go to the front, though there were women on the hospital ships. The procedure was for the men to be taken to Dalny, where there was accommodation for some 7,000. They either rested there a while or were at once put on board the hospital ships, and conducted to Hiroshima or other towns, and thence to Tokio or elsewhere. At Hiroshima about 10,000 could be received, and at Tokio some 13,000. Lastly, in order to ensure complete recovery, they were sent away to hot springs during convalescence. Mrs. Richardson was at Tokio and Hiroshima for the greater part of her stay. Everywhere she was treated with exceptional courtesy and kindness.

#### The Japanese Nurses.

The nurses were drawn from all classes of the people. The usual salary was from 18 to 20 yen (2s.) a month, during war time. No food was provided in the hospital. Each nurse brought her own. Eight to 12 hours was the average day's work, though sometimes they were obliged to work for 24 hours continuously, and they then had 20 hours' rest. Their costume was, she said, something between the Japanese kimono and the European nurse's uniform; it is entirely white, and they wear high white caps on their heads.

The Mikado has conferred on Mrs. Richardson the Sixth Class Order of the Crown, which is the highest decoration for ladies in Japan. She has similarly been awarded the highest honour of the Japanese Red Cross Society, namely, the Order of Merit.

### Sketches of Our Hospital:—The Board Room.

BY E. MARGARET FOX.

It is a grave old room, mid-Victorian, with its straight-backed chairs set in regular stiffness with their backs against the walls; scholarly, with its rows of neatly numbered medical works in their prim book-shelves; sombre, with its borrowed light from the skylight in its roof and from its narrow windows at one end, through which no glimpses may be had of green lawns or the waving trees of the garden, but looking blankly out upon a paved and enclosed courtyard surrounded by hospital buildings.

Severely simple in its appointments and furniture, it gains, rather than loses, from this very simplicity. Uncompromising in its severity, the solid table stretches its ponderous length across quite two-thirds of the bare polished floor—a table obviously to write at, to hold minute-books and ledgers, reports and accounts, tenders and vouchers, and all the other paraphernalia of committee meetings.

Long heavy curtains fall in unbroken folds from the cornice-pole, serving rather to accentuate than to modify the straight lines of the window-frames.

The benign face of the founder, long since dead, looks out from its dark heavy frame of polished wood, and seems to regard benevolently the furtherance of his designs. Two other life-sized portraits of his contemporary workers hang there as well, and together the three likenesses fill up most of the wall on that side of the room; shoulder to shoulder, side by side in their pictured silence, as they were in their strenuous lives of effort and organised work—lives belonging to that period, now fast becoming remote, when our hospital was yet " in the beginning." All three are faces with grave intentness of purpose, with kindly, though firmly set mouths, faces of philanthropists, of men who served their generation wisely and well. To them belonged the brains that evolved the plans, the energy that executed them, the money that made the work possible. Almost too near to the generation that now is for full appreciation, the memory of our founder will gather lustre with future years, and no doubt in the time to come a presentment of that mild face, with its singular aureole of snow-white hair, will be carved in stone, or set in some stained-glass window of the hospital that is yet to be, when the phœnix of the future shall have arisen from the ashes of the past.

Beneath the three portraits hangs one unpretentious little frame, wherein are several faded ribbons and tarnished medals that, dating back to the Servian and Franco-German wars of the seventies, are eloquent of devoted and far-reaching service on the part of our nurses, even when the work was but young. The silent inspirers of courage and heroism breathe many a gentle lesson to our younger workers, who sometimes now in their early days of probation, step softly into the stern old board-room and gaze at these witnesses of the past with something like reverence not unmixed with ambition.

The present associations of our nurses with the board-room are chiefly those relating to lectures and examinations; for, though mostly used for meetings of hospital committees and various societies, it is given up to them once a week for that purpose.

Scholastic, then, is its aspect, when on those occasions, the table being moved back, rows of chairs take its place and a blackboard is introduced with a lecturer's desk and other appurtenances of the schoolroom.

But still the room does not brighten, even with the evening gas, a glowing fire, and the young faces gathered there.

It is not a place to joke in or to make merry, and the few minutes before the lecture begins seldom bubble over with frivolous chatter or irresponsible laughter.

The nurses sit talking together in subdued voices, comparing notes of the last lecture with one another, and getting ready to follow with rapid pencils the hour's instruction that will follow.

The watching eyes of the portraits on the wall seem to be on them, and the grave influence of the room holds them unconsciously in seriousness. They rise on the lecturer's entrance and re-seat themselves quietly, blank white pages speedily becoming filled by the busy pencils as they listen.

No one ever comes late to these lectures. It is a point of honour and pride with our nurses to be early; and rarely too, is there any inattention or drowsiness during the hour though an obvious effort has to be made to keep awake by those on night duty, who have been called an hour earlier than usual to attend the lecture. They all realise that attention is necessary if they are to succeed, for the same rough notes have to be copied out intelligibly and handed in for correction. Then will follow an instruction class, to explain more fully the subject of each lecture, and finally comes the examination at the end of each lecturer's course.

Then is the time, if ever, when one feels small and young and ignorant, and oh! so regretful of past neglected opportunities of acquiring knowledge, and so full of repentance for one's lack of attention, one's carelessness, one's incapacities!

The board-room on these fateful evenings takes on quite its sternest aspect, in spite of the incandescent lights, garish in their brilliance, and the many small lamps scattered among the divers little tables dotted about the room for the better isolation of the victims.

The candidates enter one by one, possessed for the most part by a nervousness altogether uncalled-for by the simple task required of them. The few idle ones have been frantically reading up for the last day or two, trying to cram into their brains in a few hours the facts they should have learned by degrees weeks ago. The faces of the industrious, who have studied far and away more subjects than they will ever be required to know, look strained and anxious. Only one or two are calm and self-possessed.

They have all been assured that the examination-paper will be quite easy, and that no such tremendously high standard will be exacted from them; but they are apprehensive all the same that the examiner will make use of his unbounded opportunities to discover their very weakest points. Even the sight of the sheets of foolscap paper ranged at ordered distances down the solemn-looking table seems to take from them what little they know. Its black surface suggests only their ignorance; the white blotting-paper absorbs all their ideas.

Typewritten papers are given round to each; the questions are read aloud, slowly and distinctly, and the fatal hour has now well arrived. Realising this, each one seizes her pen, and, summoning what knowledge and resourcefulness she may possess to her aid, plunges boldly into the fray with the courage of despair. At any rate, they know the worst now that the questions are before them, and, to the infinite relief of most, find it is not so bad as they feared.

Half-forgotten facts come leaping back to the memory they had so nearly and treacherously evaded. Some of the pens race along the paper as if they can hardly keep up with the brains that urge them on. Faces become flushed and brows knit with earnest thought as they warm to their work. Here and there a hand is silently raised for another and yet another sheet of foolscap. Except for the hurrying pens and the rapid ticking of the clock, no sound is heard in the room

The faces on the wall keep a steady watch, and the case of medals mutely beckons to success.

"Five minutes more!"

At the warning voice, heads bend lower, and those who have spent too much time over one question to the exclusion of others write feverishly on, hoping in the brief span that remains to them to answer the compulsory number in time. One or two lay down their pens with an air of relief, and read critically what they have written, with a view to correction.

Some eye with dismay the smallness of their intellectual output, and rack their brains unavailingly for further facts, which, alas! are not forthcoming.

And then the deep-toned hospital clock slowly announces the hour, and the tension is relaxed.

"Time is up; stop writing please!"

The room clears quickly; but not until they are fairly outside the door are tongues loosened and the paper discussed, with the usual comments on the obscurity of the questions and the chances of coming out at the foot of the examination-list among those who are to "attend again" next year.

The notice-board is anxiously scanned for a day or two, until the list appears, and out of all proportion to the results is the prevailing excitement. When it is at last pinned up, the name at the top belongs to a nurse who sat during the examination opposite the faces on the wall. She says, and her opinion is in no wise shaken by the banter with which it is received, that their quiet calm gaze helped her to think and to take a just view—no more and no less—of the importance of the paper she was writing. Certain it is she had been steadily industrious all through the course, to which, no doubt, her success was largely due. Yet the faces of the founder and his co-workers, with their air of earnest endeavour, perhaps, were not entirely without part or lot in the matter. Who knows? Possibly, with the least-suspected things of this earth may sometimes be curiously interwoven the mysterious personalities of the quick and the dead.

### Everybody's Opinion.

[Correspondence on all subjects is invited, but we cannot in any way be responsible for the opinions expressed by our correspondents. No communication can be entertained if the name and address of the correspondent are not given as a guarantee of good faith, but not necessarily for publication. All correspondents should write on one side of the paper only.]

### LADY DOCTORS AND MIDWIVES.

Another "C.M.B." writes: Do your correspondents "J. W." and "C.M.B.," who speak of a "crying injustice being done to a large body of deserving and struggling women," refer to the medical students who are studying their profession in the same way as "J. W." and "C.M.B." did before they obtained their certificate, or do they refer to qualified Lady Doctors? Why should the fact that some hospitals ask the patients to pay 5s. towards the expenses of the institution do any more harm to the midwife than do the students from a free school?

### NURSES AND GIFTS.

"Sister" writes: A probationer may begin her hospital career with many ups and downs, and occasionally wish herself home again, especially if her staff nurse is inclined to bully and show no consideration, but expect her to know her duties, without being taught them, as happened in my case. But there is a brighter side to these small worries, and that is, the gratitude of the patient and often the friends who visit them. It was the rule of our hospital, that nurses must not receive either gifts or money. On one occasion an old man called me to carry away some fresh eggs which he had brought for his wife, and in a loud whisper he said, "Nurse, my Missus says you have been kind to her, so take that broken egg for yourself. If you was to wrap it up in a henvelope and boil quick, it would eat nice." I thanked him as heartily as if I intended carrying out his wish, but I showed my grateful patient that there were other ways of cooking broken eggs, and she enjoyed them. On another occasion a man said to me "I know you nurses enjoy a glass of beer; now, my old woman tells me that you have been so good to her, so take this threepence, and just get yourself an extra glass." I did not explain that all nurses do not like beer, or need it, but I suggested some flowers for his old woman, and she did appreciate them when he rather awkwardly presented them, on the next visiting day. My life as a probationer was a very happy one, but not in the sense that a lady, a new member of the visiting committee suggested. She was making her first visit over the hospital and saw written in big letters, "Theatre," she remarked, "How nice for the nurses to have a theatre, I didn't know they provided such a pleasant recreation for them." How little some of the outside world know what the work of that theatre means to a nurse. We always attend that particular "place of amusement," thinking of our patients, and hoping for good results and restored health.

### "NURSES AND DRESS."

"County Superintendent" writes: It has often puzzled me why nurses should pay so little attention to their dress, and I wonder if it is because they do not realise what a difference dress makes. First, let us take uniform. Why, because a woman is a nurse, need she go about with a dress a couple of inches on the ground and the bottom of her skirt well fringed? Why should her strings (oftentimes none too clean) be worn tied in an untidy bow (save the mark) at one side of her chin, and her bonnet once, shall we say blue, now be a pale shade of grey? I wonder if nurses know of an excellent preparation, and sold by all grocers, called straw paint? Its effect is wonderful, and if the ribbon be unpicked, damped, ironed, and replaced. we have a tidy bonnet at the cost of hardly a quarter of an hour, and surely it is time well spent? Gloves seem almost discarded by the district nurse, more is the pity, for what adds more to the appearance of a trim nurse than a nice tidy pair of gloves, be they only cheap navy ones, to match the uniform. Also to my mind a muddy unbrushed cloak denotes a slovenly worker. In the matter of private clothes one can nearly always tell a nurse by the fashion in which she dresses and the way she puts on her little etceteras, which are meant to give a finish to one's appearance. I know a home now where staff nurses have actually hats and jackets which they share from year to year for holiday wear, and they all receive good salaries and have held their posts for some time. Why, oh my fellow nurses, do we not strive to make the best of ourselves and not be, when on duty, as some of us undoubtedly are, an eyesore to our superintendent, and when at home often a disgrace to the family circle which we join and the friends of days gone by? My remarks chiefly apply to district nurses, with whom I have been closely associated for many years, and I do feel so sorry to think they allow themselves to degenerate. This just because their work takes them out in all weathers and amongst the very poor, who, all the while are most susceptible to trim tidy examples.

### NURSES AND MASSAGE.

"Certificated Masseuse" writes: Some few weeks ago I read in "Everybody's Opinion" of "The Nurse and the Masseuse," and have hoped each week that some more able pen than mine would have taken up the cause before now. I allude to the practice of nurses, with only ordinary training, taking up massage. I was trained about two years ago, at much expense and trouble, in general massage, medical electricity, Nauheim treatment, etc., and I hold certificates for same. I have never posed as a nurse, and have always corrected people who addressed me as "Nurse." When I obtained my certificates, the doctors in the neighbourhood seemed pleased and have been most kind in finding me work, one doctor in particular, the consulting physician to a big firm, has called me in for all the massage required in connection with accidents in the company's service. I have been successful, and have got men to work again after they had been given up by larger institutions than mine, and have had grateful letters from the doctors, managers, and workmen. This big firm's work meant about one-fourth of my earnings. Early this year the big firm decided to have a nurse of their own, and a friend of the head doctor's was engaged as such, and to look after the men generally, instead of, as before, having to depend upon hospitals or home nursing. The day of the nurse's arrival here I had two or three men under massage treatment. This was at once stopped, and I waited a week or two, hoping each day to hear something definite from either doctor or manager, but received no intimation of any sort. I then called upon the doctor and asked him what I had done, but he told me that there was nothing wrong, and that in all my 400 treatments for the firm he had never received one complaint from either manager or men. The doctor also said that the nurse was not a masseuse, and, in his opinion, nursing and massage are two separate things, and must be done by two separate attendants; and, he added, that when massage was required for the big firm I should be asked to do it, but that good nursing might prevent some of the cases getting so complicated as to require massage. The nurse tells the manager that she can do massage, but he may not know that a separate training and certificate are required for massage. I naturally feel hurt, and am awkwardly placed, as the men do not understand the difference between nursing and massage; so perhaps I am looked upon by them as not being quite so capable as somebody else. As I told the doctor, I do not blame anyone for making one salary do in place of two; but they might have told me when they engaged a nurse that perhaps my work for them would be less, or none at all. No one is more pleased than I am for the men to have a nurse in daily attendance, for I have often wanted to do more than massage for them, but am rather backward at overstepping the bounds of professional etiquette.

### Appointments.

**No charge is made for announcements under this head, and we are always glad to receive and publish appointments. The information, to insure accuracy, should be sent from the nurses themselves, and we cannot undertake to correct official announcements which may happen to be inaccurate. It is essential that in all cases the school of training should be given.]**

Bideford Hospital.—Miss Lizzie White has been appointed matron. She was trained at the East London Hospital for Children, Shadwell, and at King's College Hospital. She has since been sister at the East London Hospital for Children.

Central London Throat and Ear Hospital, Gray's Inn Road, W.C.—Miss Macdonald has been appointed matron of the Central London Throat and Ear Hospital. She was trained at St. Thomas's Hospital, joined the Army Nursing Staff and served during the war in South Africa; on returning home she was appointed assistant matron at the British Hospital for Incurables.

Colne and Holme Fever Hospital. — Miss Edith Sime and Miss Mary Turner have been appointed charge nurses. Miss Sime was trained at the Glasgow Royal Infirmary, and was afterwards charge nurse at Ham Green Fever Hospital, Bristol. Miss Turner was trained at Manchester Royal Infirmary, and was afterwards charge nurse at the Fever Hospital, Swallow Nest, near Sheffield.

Dewsbury Joint Infectious Hospital.—Miss Margaret M. Greene has been appointed senior ward sister. She was trained at King's Lynn General Hospital, Norfolk, and has since been sister at the Croydon General Hospital; senior sister at the Dewsbury and District General Infirmary; sister of the men's landing at Salop General Infirmary, Shrewsbury; and ward sister at Lewisham Infirmary.

Hastings Union Infirmary.—Miss B. Emmeline Martin has been appointed assistant nurse. She was trained at the Children's Hospital, Temple Street, Dublin, by the Meath Workhouse Nursing Association.

Jubilee Hospital Training Ship "Cornwall." — Miss M. G. Hill, R.R.C., has been appointed nurse-matron of the Jubilee Hospital Training Ship *Cornwall*, off Purfleet. She was trained at Addenbrooke's and St. Mary's Hospitals, and held the post of staff nurse at the West London Hospital. In 1893 Miss Hill joined the Army Nursing Service, and later served five years in South Africa. She was in Ladysmith during the siege, and was subsequently awarded the Royal Red Cross. She was invalided from the service on her return from South Africa.

Montgomeryshire Infirmary, Newtown.—Miss Miller has been appointed staff nurse at the Montgomeryshire Infirmary, Newtown. Miss Miller was trained at the County Antrim Infirmary, Lisburn, where she afterwards had charge of the female wards.

Shoreditch Poor-law Infirmary.—Miss Katherine Louisa Keen has been appointed ward sister. She was trained at Manchester Royal Infirmary, and has since been sister at Sheffield Royal Infirmary, and night superintendent at North Stafford Infirmary.

Workhouse Infirmary, Strood, Rochester.—Miss G. M. M. Evans has been appointed superintendent nurse at the Workhouse Infirmary, Strood, Rochester. She was trained at St. George's Infirmary, Fulham Road, where she afterwards held the posts of night superintendent and ward sister. She was also ward sister at Croydon Infirmary, Thornton Heath. Miss Evans holds the L.O.S. and C.M.B. certificates.

### Presentations.

Miss Farmer, who has held the office of matron at the Central London Throat and Ear Hospital for a period of nearly 10 years, has resigned that position and is joining her sister in the management of a nursing home in London. On leaving the hospital Miss Farmer was presented with a silver tea and coffee service by the medical staff, and with silver ornaments by the nurses of the hospital. Miss Farmer takes with her the good wishes of the committee and all connected with the institution.

### TRAVEL NOTES AND QUERIES.

By our Travel Correspondent.

Florence in November (Ida).—Yes, it is a suitable month. The winter in Florence is considered very agreeable. Remember it is often very cold there because biting winds sweep down from the mountains, there is however a large proportion of sun, which makes it an agreeable winter resort. The cheapest route is via Dieppe, Paris, and Turin, second-class single £5 8s. 10d., second-class return about £8 10s. If you want information about accommodation there tell me how much you can afford to spend per week for board and lodging and I will give you addresses. If you do not already know Florence, it would be well to read up the subject a little, to make your visit more interesting.

**Rules in Regard to Correspondence for this Section.—All questioners must use a pseudonym for publication, but the communication must also bear the writer's own name and address as well, which will be regarded as confidential. All such communications to be addressed "Travel Correspondent, 28 Southampton Street, Strand." No charge will be made for inserting and answering questions in the inquiry column, and all will be answered in rotation as space permits. If an answer by letter is required, a stamped and addressed envelope must be enclosed, together with 2s. 6d., which fee will be devoted to the objects of "The Hospital" Convalescent Fund. Ten days must be allowed before an answer can be published.**

### Notes and Queries.

**REGULATIONS.**

**The Editor is always willing to answer in this column, without any fee, all reasonable questions, as soon as possible.**

**But the following rules must be carefully observed.**

1. **Every communication must be accompanied by the name and address of the writer.**
2. **The question must always bear upon nursing, directly or indirectly.**

**If an answer is required by letter a fee of half-a-crown must be enclosed with the note containing the inquiry.**

*First-class Medical Homes.*

(176) Will you kindly tell me the best source for me to get in touch with gentlemen of good families who wish to be placed in a high-class "home" to be looked after (either mental or physical weaknesses)? I am just starting a first-class home here and want to get in touch with good patients who can afford to pay for good attention.—*E. C. M.*

Advertisements in the medical and selected lay press (such as *Morning Post*) may secure the attention you desire. The Scientific Press is publishing shortly a small directory entitled "Medical Homes for Private Patients." This will be circulated amongst medical men and the public and it should certainly serve your purpose to appear in it.

*Testimonial.*

(177) Is it the proper thing to ask the matron of my hospital for a testimonial before I finish my training in five weeks' time?—*Inquirer.*

Wait till you have completed your training before asking for a testimonial.

*Blackheath.*

(178) My parents think of settling at Blackheath, and I would be glad to know if I could obtain work in the neighbourhood. I am a fully-trained nurse (not a midwife), and have worked for 10 years, in hospital, private nursing, and in the Army during the late war. Can you let me know, therefore, if there are openings at or near Blackheath; if it is possible to obtain any daily engagement where I could live out; if you know of any place in London where midwifery training can be had free of charge?—*Hereford.*

You must make yourself known to the doctors in and near Blackheath, and advertise. For your last question, write to the Secretary, Rural Midwives Association, 47 Victoria Street, London, S.W.

*The New Red Cross Society.*

(179) Will you kindly give some information concerning the New Red Cross Society. I am referring to its objects, etc.—*Sister.*

There was an article on "The New Red Cross Society," with an account of its aims and objects, in The Hospital for July 29.

*Thonon.*

(180) I should feel grateful if you would kindly let me know what sort of mission is conducted at Thonon called the Mission Evangelique, or to whom I should apply for any information regarding it?—*M. M.*

Write to the Mother Superior, The Mission Evangelique, Thonon, Haute-Savoie, France.

*Nursing Home.*

(181) I should be much obliged if you could give me any idea of the amount of capital required to start a nursing home of six to eight beds in the West End of London?—*E. M.*

It is impossible to estimate without more particulars, but you should have sufficient capital to pay all expenses for at least two years, irrespective of any income from your venture, after you have furnished and equipped your home. There are far too many nursing homes already, and we cannot advise anyone to start another.

*Training School.*

(182) Will you please tell me whether the —— Hospital is considered a good training school?—*I. B.*

The training school you mention is an excellent one.

*Mud Baths.*

(183) I should be much obliged if you would kindly tell me if there are any "mud baths" in England?—*A. M. V.*

They can be had at Strathpeffer Spa, Ross-shire, N.B.

**Handbooks for Nurses.**

| | Post Free. |
|---|---|
| "A Handbook for Nurses." (Dr. J. K. Watson.) ... | 5s. 4d. |
| "Nurses' Pronouncing Dictionary of Medical Terms." ... | 2s. 0d. |
| "Art of Massage." (Creighton Hale.) ... ... ... | 6s. 0d. |
| "Surgical Bandaging and Dressings." (Johnson Smith.) | 2s. 0d. |
| "Hints on Tropical Fevers." (Sister Pollard.) ... ... | 1s. 8d. |

**Of all booksellers or of The Scientific Press, Limited, 28 & 29 Southampton Street, Strand, London, W.C.**

### For Reading to the Sick.

"INASMUCH AS YE HAVE DONE IT UNTO THE LEAST OF THESE."

"How can we make Thy joy our own?—
How in our heart its seed be sown?"

"My joy is yours when in crowded street
To succour the helpless your steps are fleet,
When the children see in your human face
The sunny smile of the Father's grace,
And the heart respondeth in word and deed
To the pitying cry of one in need;—
These are the seeds whence joy shall spring—
Blossom and ripen and harvest bring:
And the sheaves be gathered, a golden store—
And the gleaners follow, for still there's more."

"O grant us this joy, dear Lord," we cried,
"In all its fulness, with Thee beside."

"Inasmuch as ye do it to these," saith He,
"Shall your fulness of joy for ever be."

When your own burden is heaviest, you can always lighten a little some other burden. At the times when you cannot see God, there is still open to you this sacred possibility, to show God; for it is the love and kindness of human hearts through which the divine reality comes home to men, whether they name it or not. Let this thought, then, stay with you: there may be times when you cannot find help, but there is no time when you cannot give help.—*George S. Merriam.*

What is the will of God? Every morning and evening we pray, "Thy Will be done"; and it would seem to be futile to pray for that the meaning of which we have no conception. . . . The will of God is righteous dealing, and love, and forbearance, and hope—forward-looking—and joy. You know what these words mean. They are not shadows. You know that, in proportion as you follow after these things, the sky is brighter above you, and in your dwellings is fulness of joy You know that the common daylight is transfigured, that the daily task is hallowed, that the familiar faces of those with whom you live shine with a lustre of beauty and peace; and why? Because you have entered into the will of God. Try it; try it only for a week.

For, as you try it, you will realise this fact above all others, that not only is every single act of self-sacrifice, of love, of kindliness, blessed in itself, in its immediate result—not only on others, but on yourself—but that every single act, however trivial and small, is not isolated and alone, but is part of a higher life, of a more perfect existence, of a loftier intellect, and a diviner love. Every single act of sacrifice is part of the great sacrifice that

"Hallowed earth and fills the skies."

Every act of love and kindliness is only possible because it is part of the divine love; nothing can exist save as the result of the existence of its perfect ideal, and the ideal of perfect existence is God. . . .

The will of God is an energising power in every heart that submits to the guidance of its gentle influences.—*Shorthouse.*

What asks our Father of His children, save
Justice and mercy, and humility.
A reasonable service of good deeds,
Pure living, tenderness to human needs;
Reverence and trust, and prayer for light to see
The Master's footprints in our daily ways.

*Whittier.*

## Figures and Tables

**Fig. 9. f1:**